# Paeonol-Loaded Cyclodextrin/Composite Hydrogel for Enhanced Transdermal Delivery and Skin Photoaging Repair

**DOI:** 10.3390/gels12080746

**Published:** 2026-08-20

**Authors:** Xinrui Chen, Yong Liu, Ruofei Zu, Wenwen Li, Xueer Wang, Xinyi Yang, Chuanji Zhu, Yuling Xu, Ziwen Xie, Hongmei Xia

**Affiliations:** College of Pharmacy, Anhui University of Chinese Medicine, Hefei 230012, China; 19012658767@163.com (X.C.); 13034049882@163.com (Y.L.); 18155609958@163.com (R.Z.); 18895507846@163.com (W.L.); 15056240705@163.com (X.W.); 15005502578@163.com (X.Y.); 19355257971@163.com (C.Z.); 18969133791@163.com (Y.X.); 15555720629@163.com (Z.X.)

**Keywords:** paeonol, skin photoaging, cyclodextrin inclusion complex, composite gel

## Abstract

Skin photoaging is closely associated with oxidative stress, inflammatory responses, and dysregulated collagen metabolism. Paeonol (Pae) possesses antioxidant and anti-inflammatory activities; however, its poor water solubility and short skin retention time limit its topical application. In this study, a transdermal delivery system based on a carboxymethyl chitosan (CMCS)/Carbomer 940 (Carb940) composite gel loaded with hydroxypropyl-β-cyclodextrin inclusion complexes of paeonol (Pae-CD) was developed. Pae-CD was prepared using an ultrasound-assisted saturated aqueous solution method, and the physicochemical properties, sustained-release behavior, transdermal permeation, antioxidant activity, and safety of Pae-CD/gel were evaluated. Furthermore, a mouse model of skin photoaging induced by combined ultraviolet A (UVA)/ultraviolet B (UVB) irradiation was established to investigate its reparative effects in vivo. The results showed that Pae-CD/gel exhibited a homogeneous three-dimensional porous structure, favorable sustained-release characteristics, enhanced skin retention capacity, and good cellular compatibility. In vivo experiments demonstrated that Pae-CD/gel markedly ameliorated ultraviolet-induced skin dryness, abnormal epidermal thickening, and dermal collagen loss. It also reduced oxidative stress and inflammatory factor levels, down-regulated matrix metalloproteinase-1 (MMP-1) and matrix metalloproteinase-3 (MMP-3) expression, and promoted the restoration of collagen type I (COL-1) and hydroxyproline (HYP) levels. Systemic safety evaluation revealed no obvious toxicity. In summary, Pae-CD/gel exerts antioxidant and anti-inflammatory effects and regulates collagen metabolism by enhancing transdermal delivery and local retention, thereby providing a safe and effective topical delivery strategy for the repair of skin photoaging.

## 1. Introduction

Skin photoaging is a chronic and cumulative process of skin damage caused by prolonged or repeated UV irradiation. It is mainly characterized by wrinkle formation, skin laxity, hyperpigmentation, roughness, dryness, and impaired barrier function [1,2]. Long-term UV exposure can also induce structural alterations in the epidermis and dermis, including epidermal thickening, reduction in dermal collagen fibers, abnormal deposition of elastic fibers, and ECM remodeling, and is associated with an increased risk of photodamage-related diseases such as actinic keratosis and cutaneous malignancies [3,4]. According to wavelength and penetration depth, UVA mainly acts on the dermis, whereas UVB primarily affects the epidermis. Both can induce excessive ROS production in skin cells, thereby triggering oxidative stress injury [5]. Excessive ROS can further activate inflammation-related signaling pathways such as nuclear factor kappa-B (NF-κB) and promote the release of pro-inflammatory cytokines, includinginterleukin-1β (IL-1β), interleukin-6 (IL-6), and tumor necrosis factor-α (TNF-α) [6]. Meanwhile, ROS can also upregulate the expression of MMPs, promote collagen degradation, and inhibit collagen synthesis, ultimately leading to an imbalance in ECM metabolism and driving the development of photoaging-related skin phenotypes [7].

Pae, a natural phenolic active compound isolated from the traditional Chinese medicines Paeonia suffruticosa Andr. and Cynanchum paniculatum, exhibits well-defined multi-target pharmacological activities, including antioxidant, anti-inflammatory, and MMP-inhibitory effects [8,9]. Existing studies have demonstrated that Pae exerts significant anti-photoaging effects by scavenging free radicals, restoring endogenous antioxidant enzyme activity, blocking inflammatory cascades, and regulating collagen metabolism, indicating its considerable potential for clinical application [10]. However, the inherent physicochemical limitations of Pae, including poor water solubility, weak photostability, low transdermal permeation efficiency, and volatility at room temperature, result in low local effective concentrations and short duration of action after topical administration, which severely restrict the development and clinical translation of Pae-based topical delivery systems [11].

Cyclodextrin (CD) possesses a unique cone-shaped cavity structure with a hydrophobic interior and hydrophilic exterior. It can form host–guest inclusion complexes with lipophilic drug molecules through non-covalent interactions such as van der Waals forces and hydrogen bonding, and is therefore a classical strategy for improving the physicochemical properties of poorly soluble drugs [12]. Among CD derivatives, hydroxypropyl-β-cyclodextrin (HP-β-CD) exhibits high water solubility, low hemolytic toxicity, and good biocompatibility, and its cavity size is highly compatible with the molecular structure of Pae [13]. Previous studies have shown that HP-β-CD inclusion technology can significantly improve the water solubility and photostability of Pae while reducing its volatility [14]. However, simple aqueous solutions of Pae-CD still face challenges during topical application, including short skin retention time and susceptibility to removal by sweat, making it difficult to achieve sustained and long-acting local delivery and thereby limiting their continuous reparative efficacy in photoaged skin [15,16].

A variety of topical delivery carriers have been developed to overcome the inherent drawbacks of Pae, including liposomes, nanoemulsions, microcapsules, and single cyclodextrin inclusion complexes, yet each delivery system possesses unavoidable limitations. Although liposomes and nanoemulsions can promote skin penetration to a certain extent, they involve complicated preparation procedures and are prone to aggregation and stratification during long-term storage, resulting in unsatisfactory formulation stability. Microcapsule-based drug delivery systems fail to achieve controllable drug release rates and steady and long-term drug release [16,17]. Single cyclodextrin inclusion complexes merely improve the water solubility and photostability of Pae; lacking an adhesive skeleton on the skin surface, they are easily washed away by sweat, thereby shortening the local retention time and failing to sustain therapeutic effects. To date, the combination of cyclodextrin inclusion solubilization technology with CMCS/Carb940 composite hydrogel matrix for synchronized controlled release and long-term skin retention of Pae has not been reported. To the best of our knowledge, this study is the first to construct an integrated transdermal delivery system that combines cyclodextrin inclusion solubilization with composite hydrogel long-term retention strategies. A comprehensive systematic evaluation covering formulation physicochemical properties, in vitro skin permeation, cellular biosafety, and in vivo therapeutic efficacy is performed using a UVA/UVB-combined mouse photoaging model. This study fills the research gap of existing topical formulations that cannot simultaneously achieve favorable solubility, sustained-release performance, and skin retention capacity.

Polymeric gels, owing to their good biocompatibility, water-retaining capacity, and skin adhesion, can form a moisturizing barrier on the skin surface and are commonly used carriers for long-acting local drug delivery [18]. CMCS, a hydrophilic derivative of chitosan, possesses good biodegradability, biocompatibility, and a certain degree of free radical scavenging capacity [19,20]. Carb940 is a commonly used anionic gel matrix with strong thickening, gel-forming, and adhesive properties [21]. After CMCS and Carb940 are combined, they can form a relatively stable composite gel network through hydrogen bonding, electrostatic interactions, and molecular chain entanglement [22]. This network structure helps overcome the insufficient mechanical stability or adhesive performance of single-gel systems and endows the system with favorable drug-loading, sustained-release, and sweat-resistant properties [23]. Meanwhile, the biological activity and moisturizing characteristics of CMCS may also synergize with the antioxidant and anti-inflammatory effects of Pae, thereby jointly promoting the repair of photoaged skin [24].

**Figure 1:**
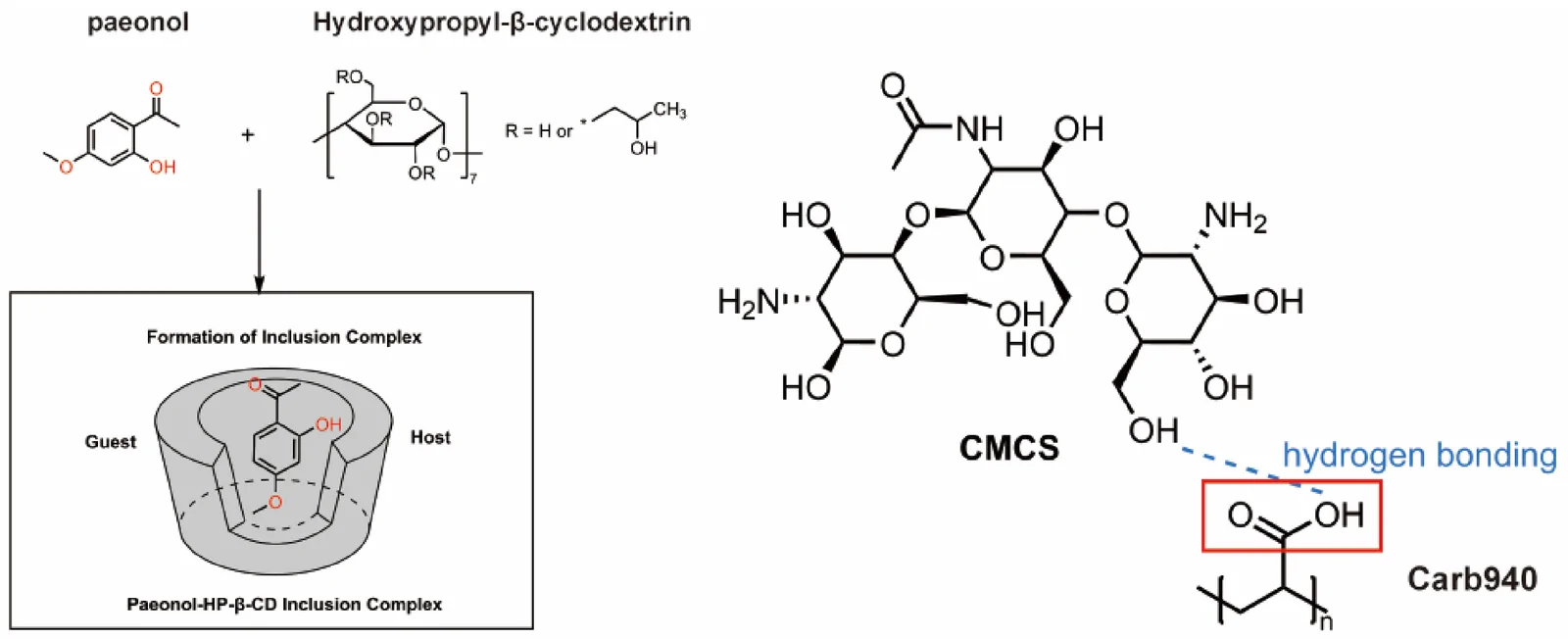
Schematic illustration of the key supramolecular interactions in this study. (**Left**) Host–guest inclusion complex formation between paeonol (Pae) and HP-β-CD to form Pae-CD. (**Right**) Hydrogen bonding interactions between CMCS and Carb940 molecular chains for constructing composite hydrogel networks.

Based on this, the present study organically combined HP-β-CD inclusion complexation solubilization technology with a CMCS/Carb940 composite hydrogel sustained-release delivery system to construct a novel transdermal delivery system, namely Pae-CD/gel, aiming to simultaneously overcome the dual drawbacks of poor water solubility and short skin retention time. A comprehensive evaluation system was established in a stepwise manner. Physicochemical characterization of the inclusion complex was performed through particle size, polydispersity index (PDI), zeta potential of the dispersion, as well as X-ray diffraction (XRD), differential scanning calorimetry (DSC), and Fourier-transform infrared spectroscopy (FTIR) analyses. The delivery performance of the formulation was evaluated by in vitro release and Franz diffusion cell transdermal experiments. Relying on a UVA/UVB-induced chronic photoaging mouse model, the in vivo repair efficacy was investigated at multiple levels by combining indicators related to skin histomorphology, oxidative stress, inflammatory cytokines, and collagen metabolism. Meanwhile, safety assessment was conducted through cytotoxicity, blood biochemistry, and organ histopathology examinations. Based on the above studies, the multi-target mechanism of Pae-CD/gel—“antioxidation, anti-inflammation, and collagen remodeling”—was further elucidated, with the goal of providing a safe and highly effective new formulation strategy for the topical treatment of skin photoaging (Figure 1).

## 2. Results and Discussion

### 2.1. Optimization of the Preparation Process of Pae-CD

**Figure 2:**
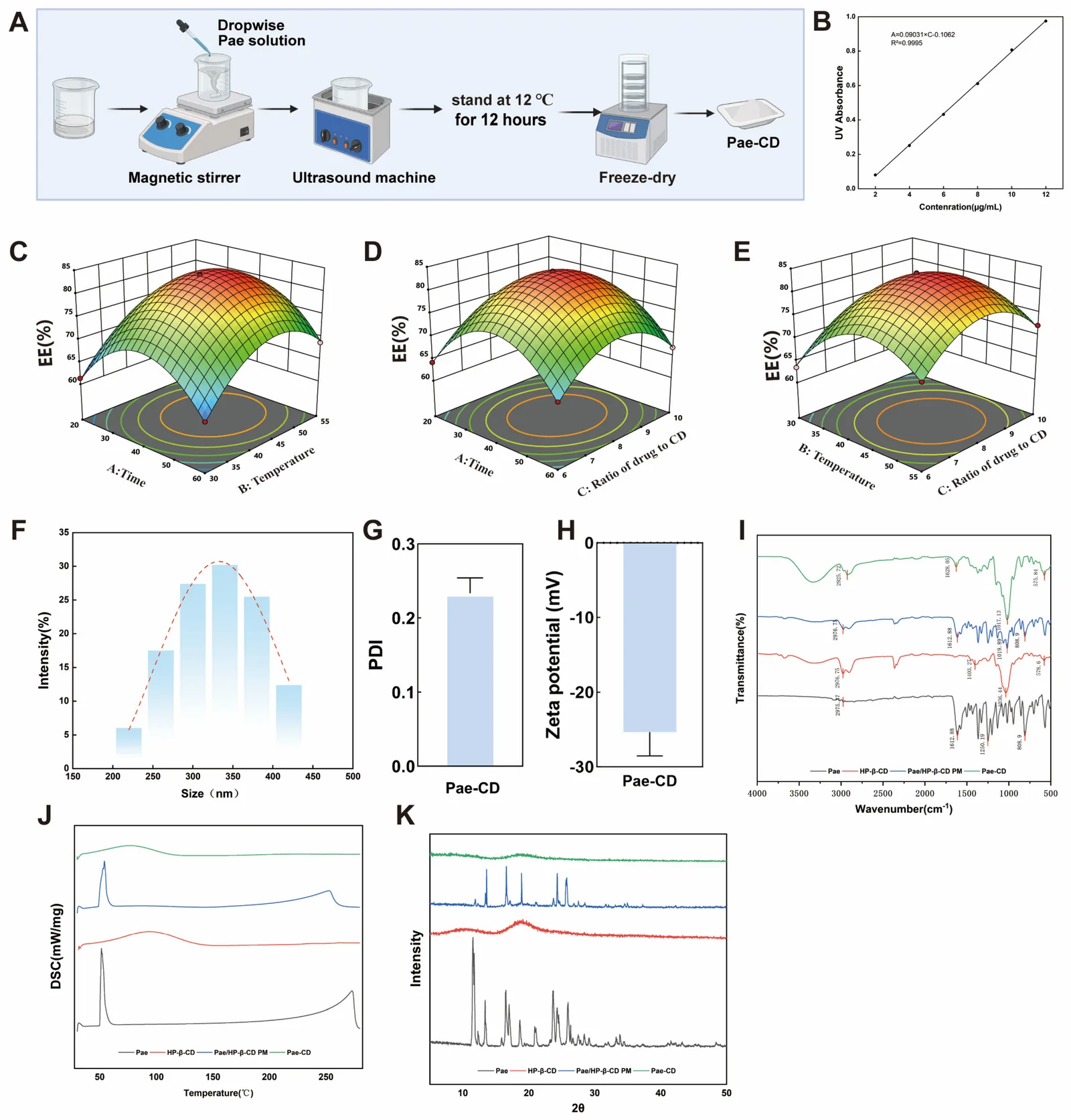
Preparation, optimization, and structural characterization of the paeonol/HP-β-cyclodextrin inclusion complex (Pae-CD). (**A**) Schematic illustration of the fabrication process of Pae-CD complexes. (**B**) UV standard calibration curve for paeonol quantification (R^2^ = 0.9995). (**C**–**E**) Three-dimensional response surface methodology (RSM) plots showing the interactive effects of inclusion time, reaction temperature, and drug-to-cyclodextrin mass ratio on encapsulation efficiency (EE, %). (**F**) Particle size distribution of Pae-CD measured by dynamic light scattering (DLS). (**G**) Polydispersity index (PDI) of Pae-CD. (**H**) Zeta potential of Pae-CD. (**I**) Fourier-transform infrared (FT-IR) spectra, (**J**) differential scanning calorimetry (DSC) thermograms, and (**K**) X-ray diffraction (XRD) patterns of free paeonol (Pae), pure HP-β-CD, Pae/HP-β-CD physical mixture (PM), and Pae-CD inclusion complex.

The preparation process of the Pae-CD inclusion complex is shown in Figure 2A.

To achieve optimal encapsulation, a UV absorbance standard curve of Pae was first established (Figure 2B) and used as the basis for quantitative analysis. Subsequently, Box–Behnken design (BBD) was employed, with ultrasonic temperature (A), ultrasonic time (B), and the mass ratio of Pae to HP-β-CD (C) selected as independent variables, and encapsulation efficiency (EE%) used as the response value (Y). A quadratic polynomial regression equation was established through multiple regression fitting as follows:

Y = 82.83 − 0.1894A − 0.2609B − 0.1801C − 0.0250AB − 6.34A^2^ − 5.56B^2^ − 4.92C^2^

The three-dimensional response surface plots (Figure 2C–E) visually demonstrated the interactive effects of the three factors on EE%. After optimization, the optimal preparation process was determined as follows: Pae: HP-β-CD mass ratio of 1:8, ultrasonic time of 40 min, and ultrasonic temperature of 40 °C. Under these conditions, three batches of parallel validation experiments were performed. The average EE% was 82.98 ± 1.21%, and the average drug loading was 9.40 ± 0.13%. The experimental values were highly consistent with the predicted values of the model, indicating that the optimized process exhibited good reliability, stability, and reproducibility.

### 2.2. Physicochemical Properties and Structural Characterization of Pae-CD

The Pae-CD prepared under optimized conditions exhibited favorable physicochemical properties. DLS analysis demonstrated that Pae-CD formed homogeneous supramolecular nano-aggregates in aqueous solution, with an average hydrodynamic diameter of 338.4 ± 12.6 nm (Figure 2F) and a low polydispersity index (PDI = 0.228 ± 0.021, Figure 2G). Although the diameter of a single HP-β-CD molecule is only approximately 1.5–2.0 nm, the formed Pae/HP-β-CD inclusion complexes can spontaneously assemble into stable nano-aggregates via intermolecular hydrogen bonding and hydrophobic interactions. In addition, the relatively negative zeta potential (−25.2 ± 3.1 mV, Figure 2H) generates strong electrostatic repulsion, ensuring excellent physical stability of this colloidal dispersion system.

To further verify whether Pae was successfully incorporated into the HP-β-CD cavity, ATR-FTIR, DSC, and XRD were performed to characterize the structure of Pae-CD using free Pae, HP-β-CD, and the their physical mixture (PM) as controls. The ATR-FTIR results showed that Pae exhibited a distinct C=O stretching vibration peak near 1612 cm^−1^, whereas in Pae-CD, this characteristic peak showed a red shift and was markedly weakened (Figure 2I). This change suggests that functional groups such as the carbonyl group in Pae may form non-covalent interactions, including hydrogen bonding and van der Waals forces, with the HP-β-CD cavity or the cavity rim region. The DSC results showed that Pae exhibited a distinct endothermic melting peak near 51.7 °C, consistent with the melting point characteristics of a crystalline drug, whereas HP-β-CD showed no sharp melting peak. The characteristic endothermic peak of Pae was still observed in the PM, while this peak was markedly weakened or disappeared in Pae-CD (Figure 2J), indicating that the original crystalline structure of Pae was disrupted and that Pae may exist in a molecular or highly dispersed state within the CD system. The XRD results further confirmed these findings. Free Pae displayed sharp crystalline diffraction peaks at multiple 2θ positions, whereas HP-β-CD mainly exhibited amorphous diffuse peaks. Some characteristic crystalline peaks of Pae were still visible in the PM, whereas the crystalline diffraction peaks of Pae almost disappeared in Pae-CD, and the overall pattern approached the amorphous characteristics of HP-β-CD (Figure 2K). Together, these results demonstrate that Pae successfully formed an inclusion complex with HP-β-CD and transformed from a crystalline state into a molecularly dispersed or amorphous state.

### 2.3. Preparation and Formulation Screening of Pae-CD/Gel

The preparation process of Pae-CD/gel is shown in Figure 2A. First, CMCS and Carb940 solutions were prepared separately, fully swollen, mixed at different ratios, and adjusted to approximately pH 6.8 using triethanolamine to promote gel formation. Subsequently, Pae-CD was added and uniformly dispersed within the CMCS/Carb940 composite gel network to obtain the Pae-CD/gel transdermal delivery system.

**Figure 3:**
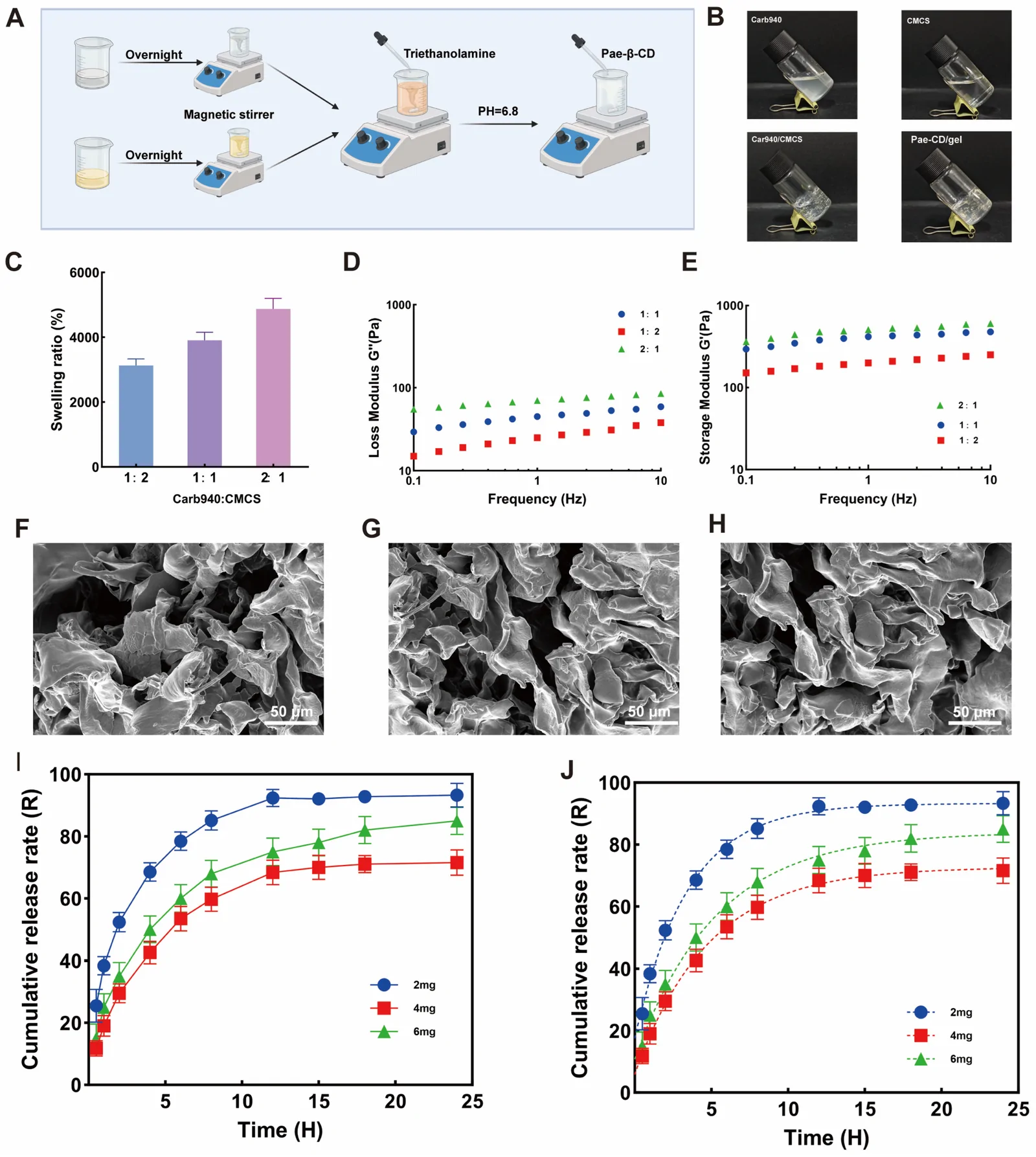
Fabrication and characterization of Pae-CD-loaded Carb940/CMCS composite hydrogels. (**A**) Schematic illustration of prepolymer blending and in situ gelation for Pae-CD-loaded hydrogels. (**B**) Digital photographs showing the macroscopic appearance of pure Carb940 gel, pure CMCS gel, blank Carb940/CMCS composite gel, and Pae-CD-loaded composite gel. (**C**) Swelling ratios of composite hydrogels with different Carb940:CMCS volumetric ratios (1:2, 1:1, and 2:1, *v*/*v*). (**D**) Loss modulus (G″) and (**E**) storage modulus (G′) of composite hydrogels with varying polymer ratios obtained from oscillatory frequency sweep tests. (**F**–**H**) Scanning electron microscopy (SEM) micrographs showing the microstructural morphology of hydrogels prepared at Carb940:CMCS volume ratios of 1:2, 1:1, and 2:1, respectively (scale bar = 50 μm). (**I**) In vitro cumulative release profiles of Pae-CD from composite hydrogels with different drug loadings (2, 4, and 6 mg/g). (**J**) Fitted kinetic curves corresponding to the in vitro release data presented in panel (**I**) for the three drug loading groups.

Different CMCS/Carb940 ratios markedly affected the appearance, swelling behavior, and microstructure of the gels. Visual observation showed that the single CMCS system exhibited weak gel-forming ability, whereas the Carb940/CMCS composite system formed a relatively stable semisolid gel. After the addition of Pae-CD, the system remained homogeneous, with no obvious phase separation or precipitation (Figure 3B). The swelling results showed that gels with different ratios all exhibited strong water absorption capacity. Among them, the gel with a CMCS-to-Carb940 volume ratio of 1:1 showed moderate and stable swelling behavior, with a 24 h swelling ratio of approximately3906 ± 244.78%, which could provide favorable moisturizing properties while avoiding excessive water uptake that might loosen the gel network or damage its structure (Figure 3C).

The viscoelastic properties of gels with different ratios were further evaluated by rheological testing. Dynamic frequency sweep results showed that G′ was higher than G″ in all gel groups over the frequency range of 0.1–10 Hz, indicating typical elastic gel characteristics (Figure 3D,E) [25,26]. Among them, the 1:1 ratio group exhibited moderate viscoelasticity, which ensured structural stability of the gel while facilitating spreading, extension, and adhesion on the skin surface. SEM results showed that the 1:1 gel presented a relatively regular and interconnected three-dimensional porous network structure with a relatively uniform pore size distribution, which was beneficial for drug loading, water absorption and swelling, and sustained-release diffusion (Figure 3F–H) [27]. Based on appearance, swelling behavior, rheological properties, and microstructure, the CMCS-to-Carb940 volume ratio of 1:1 was ultimately selected as the optimal ratio for the composite gel matrix.

After determining the optimal matrix ratio, the in vitro release profiles of Pae-CD/gel with different drug loadings were further investigated. The results revealed that the cumulative release amount of Pae increased with elevated drug loading. Nevertheless, excessively high drug loading may impair the homogeneity and release stability of the gel system. The formulation with a drug loading of 4 mg/g exhibited steady sustained release within 24 h, achieving a cumulative release rate of 71.59 ± 4.13% at 24 h without obvious initial burst release (Figure 3I,J). Although the 6 mg/g group presented a higher cumulative release percentage, analysis of hydrogel swelling behavior indicated that excessive loading of inclusion complexes disrupted the crosslinking network between CMCS/Carb940, triggering microstructural loosening and deteriorated structural stability of the three-dimensional gel framework. This reduced the diffusion resistance of the drug and accelerated drug release. In contrast, the 4 mg/g group possessed appropriate and stable swelling properties. While maintaining the structural integrity of the hydrogel matrix, it achieved more stable and controllable sustained release, which facilitated the establishment of a durable drug reservoir in the local skin. Therefore, comprehensively considering the structural stability and sustained-release performance of the gel, the final Pae concentration of 4 mg/g was selected as the optimal drug loading for Pae-CD/gel.

### 2.4. Evaluation of the In Vitro Antioxidant Activity and Cell Biosafety of Pae-CD/Gel

**Figure 4:**
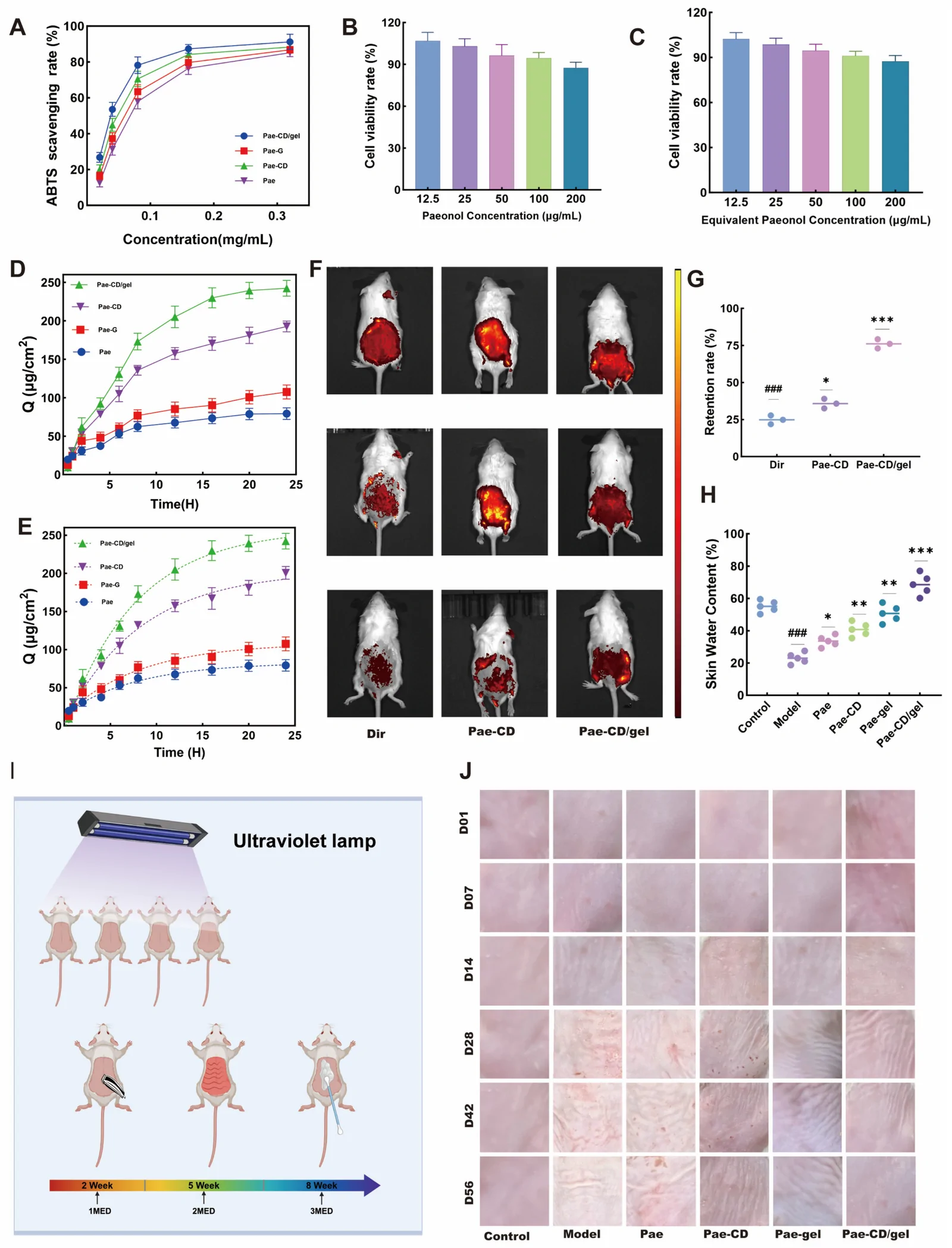
In vitro bioactivity, transdermal permeation, and in vivo anti-photoaging efficacy of Pae-CD/gel. (**A**) ABTS radical scavenging activities of free Pae, Pae-CD, Pae-gel (Pae-G), and Pae-CD/gel at gradient concentrations. (**B**,**C**) Cytocompatibility evaluation on HaCaT cells: (**B**) cell viability after incubation with serially diluted free Pae solutions; (**C**) cell viability of HaCaT cells treated with Pae-CD/gel at gradient equivalent Pae concentrations. (**D**) Twenty-four-hour cumulative transdermal permeation profiles of free Pae, Pae-CD, Pae-gel, and Pae-CD/gel measured via Franz diffusion cells. (**E**) Fitted permeation kinetic curves corresponding to the experimental data in panel (**D**). (**F**) In vivo near-infrared fluorescence imaging of mouse dorsal skin after topical administration of free DiR, DiR-labeled Pae-CD, and DiR-labeled Pae-CD/gel at predetermined time points (n = 3 per group). (**G**) Quantitative analysis of skin fluorescence retention rates derived from panel (**F**). (**H**) Dorsal skin moisture content of mice in different treatment groups (n = 5 per group). (**I**) Schematic illustration of UV-induced skin photoaging model establishment and therapeutic schedule. (**J**) Macroscopic observation of dorsal skin lesions at different time points during photoaging induction and treatment. (* *p* < 0.05, ** *p* < 0.01, *** *p* < 0.001 vs. model group; ^###^ *p* < 0.001 vs. control group).

The results of the ABTS radical scavenging assay demonstrated that both Pae-CD and Pae-CD/gel retained the intrinsic antioxidant activity of Pae. The radical scavenging rate of each group gradually increased with rising concentration and eventually reached a plateau. At the maximum tested concentration of 0.3 mg/mL, all groups achieved comparable maximum radical scavenging capacity. Nevertheless, Pae-CD/gel exhibited a lower half maximal inhibitory concentration (IC_50_) value, indicating that a lower drug concentration was required to attain equivalent radical scavenging effects compared with free Pae (Figure 4A). These findings suggest that HP-β-CD inclusion significantly improves the aqueous dispersibility of Pae, reduces drug aggregation and precipitation, and increases the proportion of available drug molecules participating in radical scavenging reactions within the system, thereby elevating the apparent antioxidant utilization efficiency of Pae.

The in vitro biosafety of Pae and Pae-CD/gel in HaCaT cells was evaluated using the CCK-8 assay. The results showed that after HaCaT cells were co-incubated with Pae and Pae-CD/gel extracts for 24 h, the relative cell viability remained above 80% at all tested concentrations (Figure 4B,C). These findings indicate that Pae and Pae-CD/gel exhibited no obvious cytotoxicity toward HaCaT cells and had good in vitro cytocompatibility.

### 2.5. In Vitro Transdermal Permeation Behavior and Skin Retention Capacity of Pae-CD/Gel

The in vitro transdermal permeation behavior of Pae from different formulations was compared using the Franz diffusion cell method. The results showed marked differences in the cumulative permeated amount among the formulations (Figure 4D,E). Free Pae exhibited a relatively low cumulative permeated amount due to its poor water solubility and certain volatility. Although the Pae-gel group could prolong drug residence on the skin surface, the porous structure of the gel imposed a certain barrier to drug diffusion, resulting in relatively limited release and permeation rates. In contrast, the Pae-CD/gel group exhibited superior transdermal delivery capacity, with a higher 24 h cumulative permeated amount than the Pae, Pae-CD, and Pae-gel groups.

DiR fluorescence tracing was further used to evaluate the skin retention capacity of the formulations under simulated sweat-rinse conditions. In vivo imaging showed that after artificial sweat spraying and standing, the skin fluorescence signals in the free DiR solution group and Pae-CD-DiR solution group were markedly attenuated, whereas a strong and relatively intact fluorescence signal remained visible in the administration area of the Pae-CD/gel-DiR group (Figure 4F). Quantitative analysis showed that the skin fluorescence retention rates of the free DiR and Pae-CD-DiR groups were 24.87 ± 2.98% and 35.78 ± 3.22%, respectively, whereas that of the Pae-CD/gel-DiR group reached 76.12 ± 3.11%, which was significantly higher than those of the solution groups (Figure 4G). These results indicate that the composite gel matrix significantly enhanced the skin adhesion and sweat-rinse resistance of the formulation, thereby facilitating prolonged local action of Pae.

### 2.6. Improvement Effects of Pae-CD/Gel on the Appearance and Moisture Content of UVA/UVB-Induced Photoaged Mouse Skin

The construction process of the UVA/UVB-induced mouse photoaging model is shown in Figure 4I. Except for the blank control group, all mice were subjected to increasing doses of UVA/UVB irradiation for 8 weeks and received different formulation interventions starting from week 3. As the irradiation period progressed, the dorsal skin of mice in the UV model group gradually developed typical photoaging manifestations, including erythema, dryness, desquamation, roughness, and deepened wrinkles [28]. At week 8, skin damage in the model group was most pronounced, characterized by disordered skin texture, reduced elasticity, and leather-like changes. Compared with the model group, all treatment groups alleviated UV-induced skin damage to varying degrees, with the Pae-CD/gel group showing the most prominent improvement. In this group, erythema and desquamation of the dorsal skin were markedly reduced, wrinkle severity was decreased, and the skin surface became relatively smooth, with an overall appearance closer to that of the normal control group (Figure 4J). These findings suggest that Pae-CD/gel can effectively alleviate UVA/UVB-induced skin photoaging phenotypes.

The results of skin moisture content measurement further showed that UV irradiation significantly reduced skin moisture content in the model group, indicating impairment of the skin barrier and moisturizing capacity. Different treatment groups increased skin moisture content to varying degrees, among which the Pae-CD/gel group showed the most obvious improvement (Figure 4H). This result indicates that the CMCS–Carb940 composite gel not only serves as a sustained-release drug carrier but also forms a moisturizing adhesive layer on the skin surface, thereby improving the water-retention capacity and barrier status of photoaged skin.

### 2.7. Reparative Effects of Pae-CD/Gel on the Histopathological Structure of Photoaged Skin

**Figure 5:**
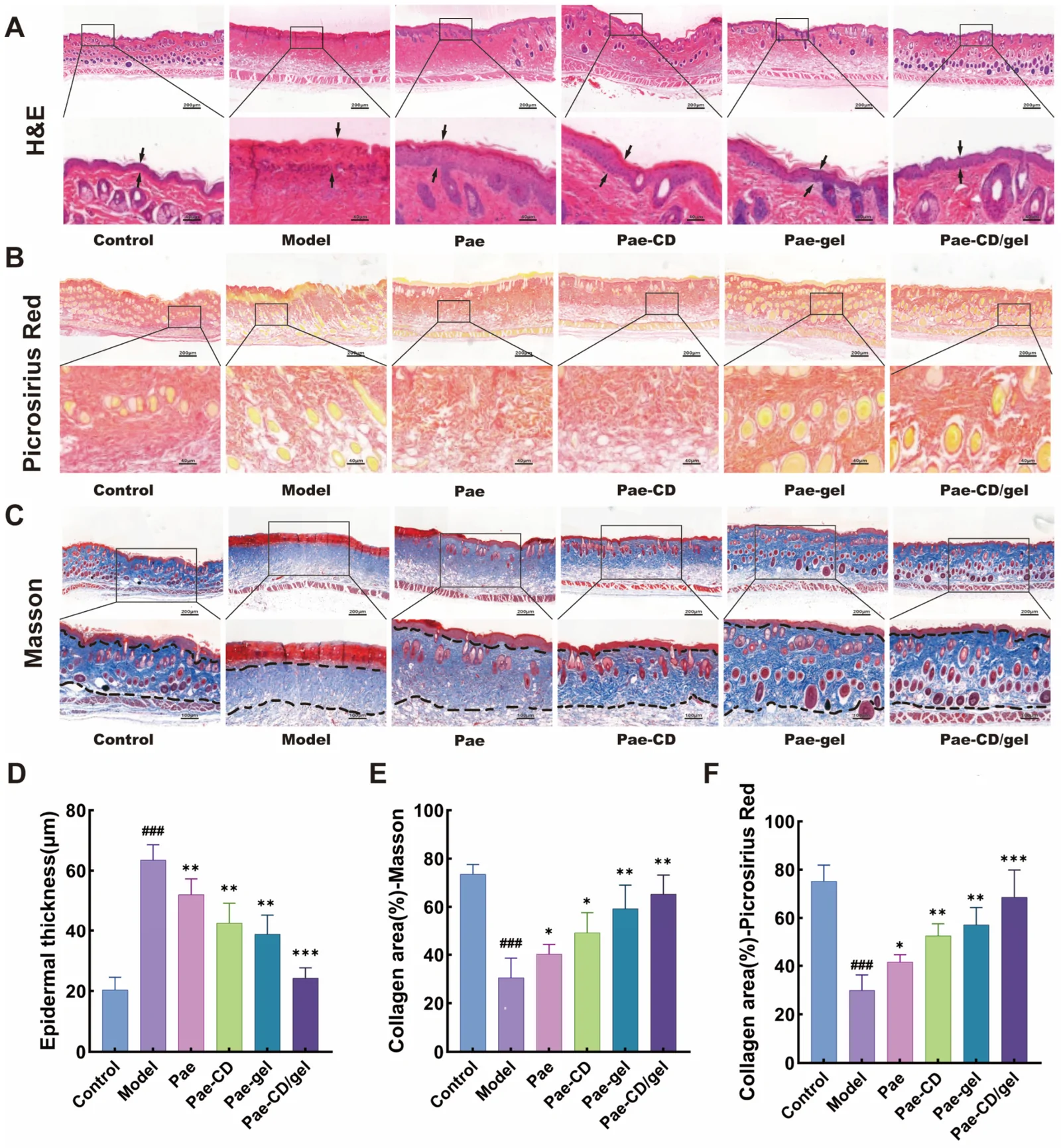
Histopathological staining and quantitative morphological analysis of mouse skin tissues across different treatment groups. (**A**) Representative hematoxylin and eosin (H&E) staining of dorsal skin sections, with high-magnification images detailing epidermal morphology and structural alterations (arrows denote epidermal layer differences). (**B**) Representative Picrosirius Red staining depicting the distribution and abundance of dermal collagen fibers. (**C**) Representative Masson’s trichrome staining visualizes dermal collagen deposition (collagen fibers stain blue; dashed lines in high-magnification views delineate the boundary). (**D**) Quantitative measurement of epidermal thickness derived from H&E-stained sections. (**E**) Quantitative analysis of collagen-positive area ratio based on Masson’s trichrome staining. (**F**) Quantitative analysis of collagen-positive area ratio based on Picrosirius Red staining. (* *p* < 0.05, ** *p* < 0.01, *** *p* < 0.001 vs. model group; ^###^ *p* < 0.001 vs. control group).

To further evaluate the reparative effects of Pae-CD/gel on the tissue structure of photoaged skin, hematoxylin and eosin (H&E), Masson’s trichrome, and Picrosirius Red staining were performed to observe epidermal thickness, inflammatory infiltration, and dermal collagen changes (Figure 5A–C). H&E staining showed that the epidermis in the normal group was thin and continuous, with a clear dermal structure. In contrast, the UV model group exhibited marked epidermal thickening, abnormal keratinization, and dermal inflammatory cell infiltration. Quantitative analysis showed that epidermal thickness in the model group significantly increased from 20.41 ± 4.19 μm in the normal group to 63.55 ± 5.11 μm. After intervention with different formulations, abnormal epidermal thickening was alleviated to varying degrees. Among them, the epidermal thickness in the Pae-CD/gel group decreased to 24.29 ± 3.39 μm, which was significantly lower than that in the model group (Figure 5D). These results indicate that Pae-CD/gel can markedly inhibit UV-induced compensatory epidermal thickening and abnormal keratinization.

To more precisely observe the density and arrangement of collagen fibers in the dermis, Sirius Red staining (Figure 5B) and Masson’s trichrome staining (Figure 5C) were performed. The staining images and the corresponding quantitative analysis of collagen area percentage (Collagen area, %) (Figure 5E,F) showed highly consistent trends. In the model group, collagen fibers in the dermis were disorganized, and the collagen area percentage decreased to approximately 30%. After treatment with Pae-CD/gel, dense, strongly stained, and highly orderly blue/red collagen fiber bundles were observed in the dermis, accompanied by a marked recovery in collagen area percentage. This dual histopathological evidence provides strong morphological confirmation that Pae-CD/gel effectively remodels the microscopic architecture of the skin.

### 2.8. Regulatory Effects of Pae-CD/Gel on Collagen Metabolic Balance in Photoaged Skin

**Figure 6:**
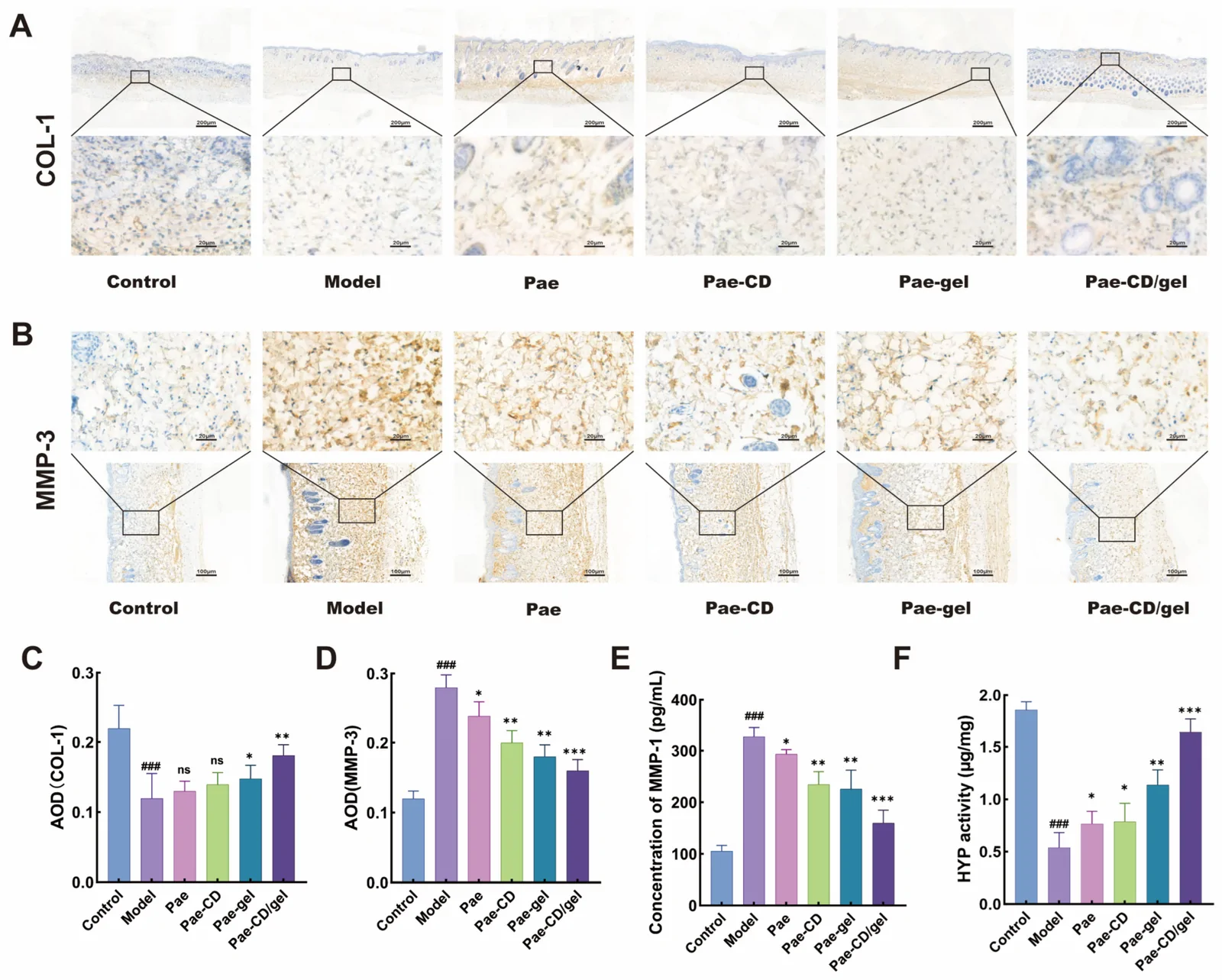
Reparative effects of Pae-CD/gel on collagen loss in the skin of photoaged mice. (**A**) Immunohistochemical staining of COL-1 in dorsal skin sections, with magnified views showing positive expression in the dermal layer. (**B**) Immunohistochemical staining of MMP-3 in dorsal skin sections of each group. (**C**) Quantitative analysis of AOD of COL-1 positive staining. (**D**) Quantitative analysis of the AOD of MMP-3 positive staining. (**E**) Tissue concentration of MMP-1 in skin homogenates. (**F**) HYP content in skin tissues, reflecting total collagen deposition. (* *p* < 0.05, ** *p* < 0.01, *** *p* < 0.001 vs. model group; **^###^** *p* < 0.001 vs. control group; ns: not significant).

To further investigate the possible mechanism by which Pae-CD/gel promotes dermal collagen repair, this study measured key indicators related to ECM metabolism, including COL-1, MMP-3, MMP-1, and HYP (Figure 6). Immunohistochemical results showed that COL-1 expression was strongly positive in the dermis of the normal group, whereas the positive staining intensity of COL-1 was markedly reduced in the UV model group, suggesting that combined UVA/UVB irradiation inhibited type I collagen deposition. After Pae-CD/gel treatment, the positive staining intensity of COL-1 in the dermis was obviously enhanced (Figure 6A). Quantitative analysis revealed that the COL-1 AOD value in the model group was significantly lower than that in the normal group, while the COL-1 average optical density (AOD) value in the Pae-CD/gel group was significantly higher than that in the model group (Figure 6C), indicating that Pae-CD/gel effectively restored the expression level of type I collagen in photoaged skin.

The immunohistochemical results for MMP-3 showed that the positive expression of MMP-3 in the dermis of the UV model group was significantly enhanced compared with the normal group, demonstrating that UV irradiation promoted the expression of ECM degradation-related factors. After Pae-CD/gel intervention, the positive staining intensity of MMP-3 was markedly weakened (Figure 6B), and quantitative analysis further confirmed that the MMP-3 AOD value in the Pae-CD/gel group was significantly lower than that in the model group (Figure 6D). ELISA results also showed that the MMP-1 level in the model group was significantly elevated compared with the normal group, whereas Pae-CD/gel intervention significantly reduced the MMP-1 content (Figure 6E). Both MMP-1 and MMP-3 are key proteases involved in collagen degradation and ECM remodeling. The synchronous downregulation of their expression levels suggests that Pae-CD/gel may mitigate UV-induced dermal ECM damage by inhibiting MMP-mediated ECM degradation.

Regarding collagen synthesis, the HYP content assay showed that the HYP level in the UV model group was significantly reduced compared with the normal group, indicating a decrease in total skin collagen content. After Pae-CD/gel treatment, the HYP content rebounded significantly (Figure 6F). Taken together, the results of COL-1, MMP-1, MMP-3, and HYP indicate that Pae-CD/gel may correct the ECM metabolic imbalance in photoaged skin by inhibiting MMP-mediated ECM degradation and promoting collagen deposition, thereby facilitating the structural repair of the dermal ECM network.

### 2.9. Regulatory Effects of Pae-CD/Gel on Inflammatory Responses and Oxidative Stress in Photoaged Skin

**Figure 7:**
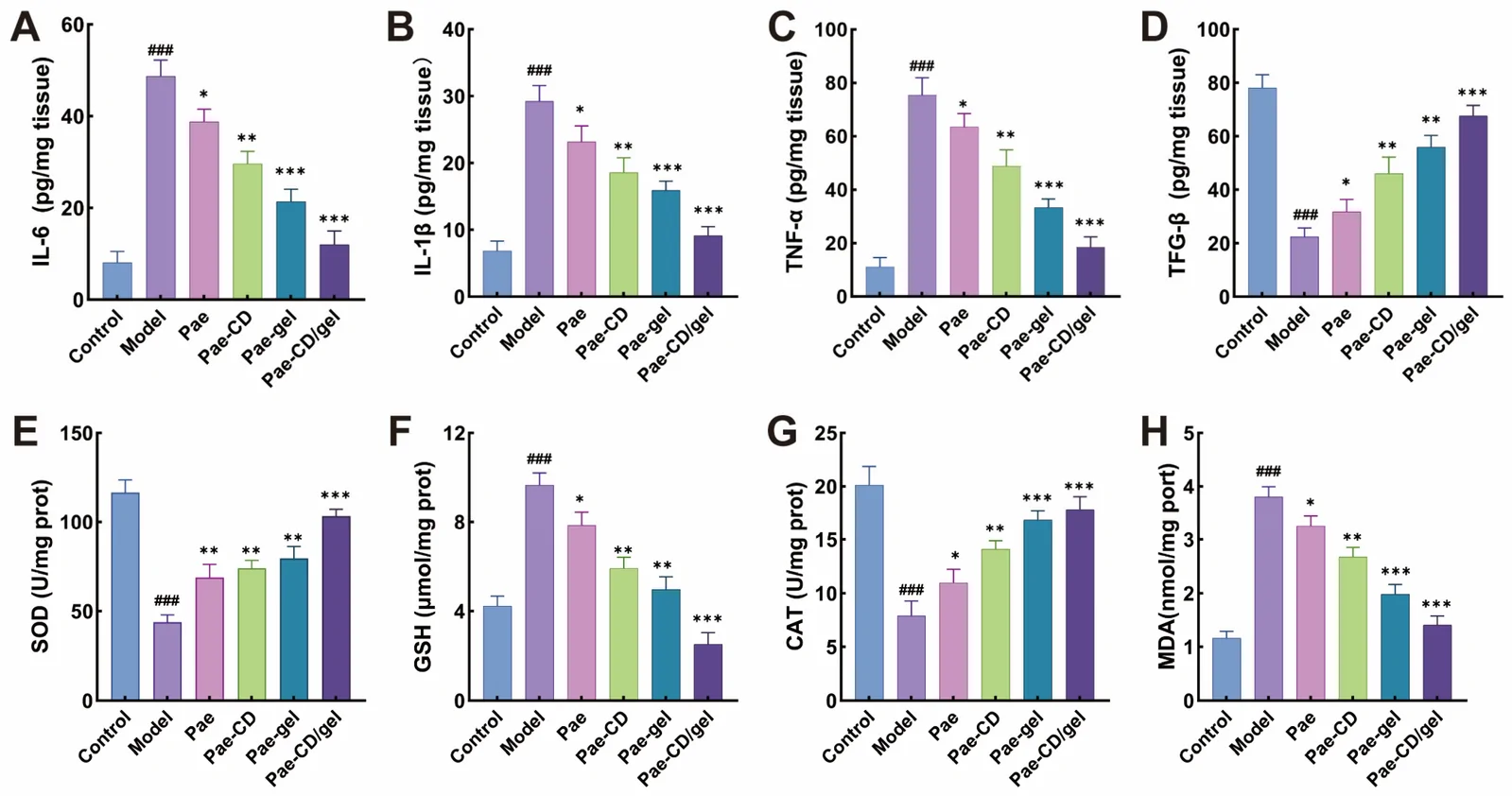
Effects of different formulations on inflammatory cytokines and oxidative stress indicators in UV-induced photoaged mouse skin. (**A**–**D**) Levels of inflammatory cytokines in skin homogenates: (**A**) IL-6, (**B**) IL-1β, (**C**) TNF-α, and (**D**) TGF-β. (**E**–**H**) Oxidative stress-related biochemical parameters in skin tissues: (**E**) SOD, (**F**) GSH, (**G**) CAT, and (**H**) MDA. (* *p* < 0.05, ** *p* < 0.01, *** *p* < 0.001 vs. model group; ^###^ *p* < 0.001 vs. control group).

UV irradiation can induce marked inflammatory responses and oxidative stress injury in skin tissues. In this study, enzyme-linked immunosorbent assay (ELISA) and biochemical assay kits were used to detect inflammatory factors and oxidative stress-related indicators in skin tissues (Figure 7). The results of inflammatory factor detection showed that the levels of IL-6, IL-1β, and TNF-α in the skin tissues of the UV model group were significantly increased, indicating that long-term UVA/UVB irradiation induced an obvious inflammatory cascade (Figure 7A–C). After treatment with different formulations, the levels of these pro-inflammatory factors decreased to varying degrees, with the most pronounced reduction observed in the Pae-CD/gel group. These results demonstrate that Pae-CD/gel can significantly inhibit UV-induced inflammatory factor release and improve the chronic inflammatory microenvironment of photoaged skin.

Transforming growth factor-beta (TGF-β) is an important factor regulating fibroblast function and collagen synthesis. The results showed that TGF-β levels were markedly decreased in the UV model group, whereas TGF-β content was significantly restored after Pae-CD/gel treatment (Figure 7D). Combined with the increased COL-1 expression and recovered HYP content, these findings suggest that Pae-CD/gel may enhance neocollagen synthesis capacity by promoting the recovery of TGF-β-related pathways.

The detection of oxidative stress indicators showed that superoxide dismutase (SOD) and catalase (CAT) activities, as well as glutathione (GSH) content, were significantly decreased in the UV model group, whereas malondialdehyde (MDA) levels were significantly increased. These results indicate that UV irradiation disrupted the endogenous antioxidant defense system of the skin and induced lipid peroxidation injury [29] (Figure 7E–H).

### 2.10. Evaluation of the In Vivo Systemic Safety of Pae-CD/Gel

**Figure 8:**
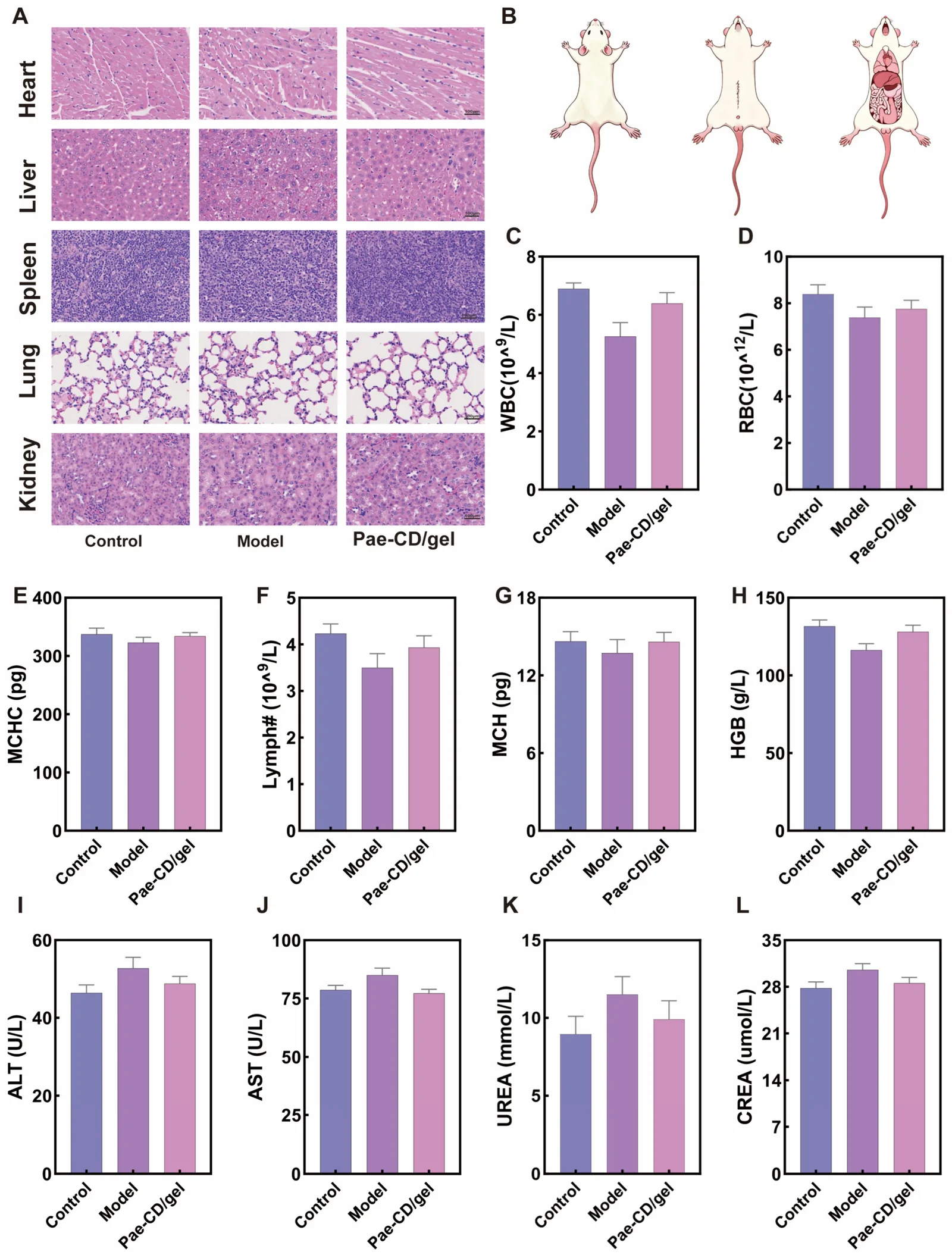
In vivo biosafety evaluation of the Pae-CD/gel composite hydrogel. (**A**) H&E-stained sections of major organs (heart, liver, spleen, lung, and kidney) from the control, model, and Pae-CD/gel groups; no obvious histopathological abnormalities were observed. (**B**) Schematic illustrations showing the gross appearance and visceral anatomical state of mice at the end of the experimental period. (**C**–**H**) Routine hematological parameters: (**C**) WBC, (**D**) RBC, (**E**) MCHC, (**F**) Lymph#, (**G**) MCH, and (**H**) HGB. (**I**–**L**) Serum biochemical markers for hepatic and renal function assessment: (**I**) ALT, (**J**) AST, (**K**) UREA, and (**L**) CREA.

To evaluate the systemic safety of Pae-CD/gel during continuous topical administration, the morphology of major organ tissues, routine blood parameters, and liver and kidney function indicators in mice were examined after the experiment (Figure 8). H&E staining of major organs showed that the structures of the heart, liver, spleen, lung, and kidney tissues in the Pae-CD/gel group remained intact, with no obvious cellular degeneration, necrosis, inflammatory infiltration, or tissue structural destruction (Figure 8A). Routine blood test results showed that WBC, RBC, HGB, MCH, MCHC, lymphocytes, and other parameters in the Pae-CD/gel group exhibited no obvious abnormal changes compared with those in the normal control group (Figure 8C–H). Liver and kidney function tests showed that ALT, AST, UREA, and CREA levels in the Pae-CD/gel group were within the normal ranges, with no significant differences compared with the control group. These results indicate that Pae-CD/gel did not cause obvious hematological toxicity, hepatotoxicity, or nephrotoxicity at the administered dose and during the treatment period used in this study, demonstrating good in vivo systemic safety.

### 2.11. Discussion

Skin photoaging is a chronic cumulative injury process caused by long-term UV exposure, and its occurrence and progression involve multiple pathological events, including oxidative stress, inflammatory responses, skin barrier disruption, and dysregulated collagen metabolism [30]. UVA can penetrate deeply into the dermis and induce sustained ROS generation, whereas UVB mainly damages the epidermis and triggers inflammatory responses [31]. The combined effects of UVA and UVB can impair the endogenous antioxidant system, increase the release of pro-inflammatory factors, upregulate MMP expression, and inhibit collagen synthesis, ultimately leading to skin dryness, erythema, deepened wrinkles, abnormal epidermal thickening, and dermal collagen loss. Therefore, an ideal topical anti-photoaging formulation should not only possess antioxidant and anti-inflammatory activities but also exhibit favorable transdermal delivery capacity, skin retention performance, and sustained-release characteristics.

Pae is a natural phenolic compound with antioxidant, anti-inflammatory, and collagen metabolism-regulating potential. However, its poor water solubility, relatively high volatility, insufficient photostability, and short skin retention time limit its further application as a topical anti-photoaging agent [32]. To address these issues, this study employed HP-β-CD inclusion technology to improve the aqueous dispersibility of Pae and further loaded Pae-CD into a CMCS–Carb940 composite gel to construct the Pae-CD/gel transdermal delivery system. This strategy simultaneously meets the requirements of drug solubilization, sustained release, skin adhesion, and sweat-rinse resistance, thereby helping to prolong the effective local action time of Pae in the skin.

HP-β-CD possesses a cavity structure with a hydrophobic interior and hydrophilic exterior and can form inclusion complexes with hydrophobic drugs through non-covalent interactions such as hydrophobic interactions, hydrogen bonding, and van der Waals forces. In this study, ATR-FTIR results showed that the characteristic carbonyl peak of Pae was red-shifted and weakened, DSC showed that the characteristic melting peak of Pae was markedly weakened or disappeared, and XRD showed that the crystalline diffraction peaks of Pae almost disappeared. These findings indicate that the original crystalline structure of Pae was disrupted and that Pae may be dispersed in the HP-β-CD system in a molecular or amorphous state. Collectively, these results confirm the successful formation of Pae-CD. The RSM optimization results further showed that the host–guest ratio, ultrasonic temperature, and ultrasonic time all affected EE%, and that the preparation process had a clearly defined optimal range. The final encapsulation efficiency was high and stable, indicating that the process had good reproducibility and controllability.

Although Pae-CD can improve the water solubility of Pae, it may still be easily lost and show a short retention time when applied topically as an aqueous solution. Therefore, Pae-CD was further loaded into the CMCS/Carb940 composite gel in this study. CMCS exhibits good hydrophilicity, biocompatibility, and certain biological activity, whereas Carb940 has strong thickening and gel-forming abilities. After combination, the two components can form a stable three-dimensional network structure. SEM results showed that the optimized gel had an interconnected porous structure, which was beneficial for drug loading, water retention, and sustained release. Rheological results showed that G′ was higher than G″, suggesting typical elastic gel characteristics. Moderate viscoelasticity helps the formulation spread uniformly on the skin surface and form a relatively stable drug reservoir at the administration site [33]. Notably, at the formulation pH of 6.8, the composite hydrogel as a whole exhibited a distinct negative surface charge profile: the dissociation of carboxyl groups abundant in Carb940 molecules formed carboxylate anions, which, together with the ionized carboxymethyl groups carried by CMCS, predominantly governed the negative charge property of the polymeric network. Meanwhile, the loaded Pae-CD inclusion complexes themselves possessed a negative charge, thereby maintaining the intrinsic negative charge nature of the hydrogel matrix. This stable negatively charged network not only conferred excellent colloidal electrostatic repulsion stability to the hydrogel but also regulated the electrostatic interactions at the hydrogel–skin interface, indirectly optimizing drug spreading and retention behaviors in the epidermis and providing a solid physicochemical foundation for its sustained transdermal delivery.

In vitro release and transdermal permeation results demonstrated that Pae-CD/gel achieved a smooth, sustained release profile and significantly enhanced the cumulative transdermal permeation amount of Pae, outperforming the free Pae-CD aqueous solution. This distinction stems from the synergistic enhanced-permeation mechanisms of HP-β-CD inclusion solubilization combined with the CMCS/Carb940 hydrogel matrix. On one hand, HP-β-CD encapsulation elevated the apparent solubility of Pae in advance, thereby increasing the free drug concentration in the donor chamber and establishing a higher baseline concentration gradient for transdermal diffusion. Although the free Pae-CD solution offered an equivalent solubilization effect, it failed to maintain a persistent osmotic driving force due to the absence of a polymeric carrier framework. On the other hand, the highly swellable 3D porous network constructed by Carb940 formed a micro-occlusive environment on the skin surface, continuously inducing stratum corneum hydration and loosening the ordered structure of intercellular lipids, which effectively reduced barrier diffusion resistance. In contrast, rapid water evaporation from the free solution resulted in only a transient and negligible permeation-enhancing effect. Meanwhile, the CMCS polymer chains—rich in polar functional groups such as hydroxyl and carboxyl groups—interacted with stratum corneum components via hydrogen bonding and electrostatic forces. This moderately loosened the barrier architecture while imparting superior skin adhesion and sweat-washout resistance to the formulation, establishing a long-lasting drug reservoir in the epidermis to sustain a stable transdermal concentration gradient over extended periods. Conversely, the free solution lacked bioadhesive components and rapidly dried or washed away with sweat after application, leading to a sharp decline in drug concentration and a subsequent collapse of the diffusion driving force. In summary, the multiple synergistic effects of the hydrogel matrix—including long-term hydration enhancement, a stable drug reservoir, and a sustained concentration gradient—constitute the key mechanism underlying the significantly superior cumulative transdermal permeation of Pae-CD/gel compared to the free Pae-CD solution.

In the UVA/UVB-induced mouse photoaging model, the model group exhibited typical injury manifestations, including obvious skin dryness, erythema, desquamation, deepened wrinkles, abnormal epidermal thickening, and dermal collagen loss. After Pae-CD/gel intervention, the appearance of the dorsal skin was markedly improved, skin moisture content was increased, epidermal thickness was reduced, and the arrangement and content of dermal collagen fibers were restored, indicating that this formulation could effectively alleviate UV-induced structural skin damage. Compared with Pae, Pae-CD, and Pae-gel, Pae-CD/gel showed a more pronounced overall improvement, suggesting a synergistic effect between inclusion-based solubilization and gel-mediated sustained release and adhesion, which enabled the anti-photoaging activity of Pae to be more fully exerted.

Oxidative stress is an important initiating factor in photoaging. UV irradiation can cause excessive ROS generation, thereby inducing lipid peroxidation, protein oxidation, and cellular structural damage. In this study, MDA levels were increased in the UV model group, whereas SOD and CAT activities and GSH content were decreased, indicating that skin tissues were under marked oxidative stress. After Pae-CD/gel treatment, MDA levels decreased, while SOD, CAT, and GSH levels were restored, suggesting that this formulation enhanced the endogenous antioxidant defense capacity of the skin and reduced lipid peroxidation injury. This effect may be attributed not only to the phenolic hydroxyl structure and free radical scavenging ability of Pae itself but also to the improved aqueous accessibility of Pae after HP-β-CD inclusion and the prolonged local action time provided by the gel.

Inflammatory responses are an important link between oxidative stress and tissue injury [34]. Excessive ROS can promote the release of pro-inflammatory cytokines, including IL-1β, IL-6, and TNF-α, thereby further aggravating skin tissue damage [35]. The results of this study showed that the levels of these inflammatory factors were elevated in the UV model group, whereas they were markedly reduced after Pae-CD/gel treatment, indicating that Pae-CD/gel effectively alleviated UV-induced inflammatory responses. The reduction in inflammatory factors not only helps attenuate skin erythema and inflammatory cell infiltration but may also indirectly reduce inflammation-associated collagen degradation, thereby protecting the dermal ECM structure [36].

Dysregulated collagen metabolism is an important pathological basis of dermal photoaging [37]. UV irradiation can promote the expression of collagen degradation-related factors such as MMP-1 and MMP-3 while inhibiting collagen synthesis- or deposition-related indicators such as COL-1, TGF-β, and HYP [38]. The results of this study showed that Pae-CD/gel reduced the expression of MMP-1 and MMP-3 and promoted the recovery of COL-1, TGF-β, and HYP levels, suggesting that it may improve ECM metabolic imbalance in photoaged skin by reducing collagen degradation and promoting collagen deposition. This finding is consistent with the collagen fiber recovery observed by Masson and Sirius Red staining, further demonstrating that Pae-CD/gel can promote dermal structural repair in photoaged skin.

Safety is a critical consideration in the development of topical formulations [39]. In this study, HaCaT cell experiments showed that Pae-CD/gel had no obvious cytotoxicity toward keratinocytes. After continuous topical administration, H&E staining of major organs showed no obvious pathological damage, and routine blood parameters as well as liver and kidney function indicators showed no marked abnormalities, indicating that Pae-CD/gel had good in vitro and in vivo biosafety at the administered dose and during the treatment period used in this study. It should be noted that this study mainly explored the mechanism of action from the perspectives of histological observation and related factor levels. Specific signaling regulatory processes, such as NF-κB, MAPK, and TGF-β/Smad pathways, have not yet been further verified. In addition, differences remain between mouse skin and the human skin barrier. Further studies using skin models more closely resembling clinical conditions, long-term stability evaluation, and investigations of drug distribution within skin layers are still needed for validation.

Pae-CD/gel effectively alleviates UV-induced skin photoaging damage and exhibits promising development prospects as a topical skin formulation; its clinical translational potential can be analyzed from multiple dimensions. Regarding dosage, the effective topical dose obtained in this study was based on a mouse dorsal skin model. The candidate human administration concentration can be preliminarily estimated based on the body surface area conversion between humans and mice. However, given the interspecies differences in skin barrier thickness and transdermal drug permeability between humans and mice, dermal irritation tests and human skin permeation studies remain necessary to establish a safe and effective clinical dosage. In terms of formulation stability, the CMCS/Carb940 aqueous composite hydrogel contains a highwater content. Systematic accelerated stability testing and long-term storage evaluations are required in future work to continuously monitor gel appearance, rheological properties, Pae content, and microbiological stability during storage, with optimization of a suitable preservative system when necessary. Concerning scalable manufacturing, the Pae-CD inclusion complex relies on stirring complexation and rotary evaporation under reduced pressure to remove organic solvents, while the preparation of the composite hydrogel involves only polymer swelling and mild homogenization mixing. The entire process operates under mild conditions, is simple to execute, and requires no complex specialized equipment. Moreover, HP-β-CD, CMCS, and Carb940 are all commercially mature pharmaceutical excipients, conferring high potential for scale-up from laboratory synthesis to pilot-scale and industrial production.

Furthermore, the limitations of this study regarding in vitro cellular experiments warrant discussion. Here, only HaCaT keratinocytes were employed to evaluate the cytocompatibility of the formulation. However, the mechanistic conclusions concerning “regulation of collagen metabolism” in this paper primarily involve the function of dermal fibroblasts. Although HaCaT cells serve as an epidermal cell model capable of verifying the formulation’s safety toward the epidermis, they cannot directly reflect its regulatory effects on collagen synthesis and degradation in dermal fibroblasts. Future studies should incorporate human dermal fibroblasts (HDFs) to establish a UVB-induced in vitro photoaging cell model, directly investigating the effects of Pae-CD/gel on the expression of COL-1 and MMP-1/3 in HDFs, while verifying the underlying regulatory mechanisms of the TGF-β Smad and MAPK/AP-1 signaling pathways via Western blot. In addition, constructing an in vitro co-culture system of HaCaT cells and HDFs to simulate epidermal–dermal crosstalk will help elucidate, in a more comprehensive manner, the indirect regulatory actions of Pae-CD/gel on dermal cell functions following transdermal delivery.

## 3. Conclusions

In this work, a novel paeonol/CMCS–Carb940 composite hydrogel for topical application (Pae-CD/gel) was successfully fabricated. This study innovatively integrates the HP-β-CD inclusion solubilization strategy and the bioadhesive hydrogel long-term skin retention delivery strategy for paeonol transdermal administration. The optimized Pae-CD inclusion complex greatly improves the water dispersibility of hydrophobic Pae. The porous interpenetrating polymer network formed by combined CMCS and Carb940 endows the hydrogel with appropriate swelling properties, favorable rheological characteristics, sustained drug release behavior, and excellent spreadability on the skin. Critically, the composite hydrogel can markedly prolong the skin residence time of the drug and resist sweat washing, overcoming the drawback of short skin retention time of conventional topical Pae preparations. In vitro cellular experiments demonstrate that Pae-CD/gel exhibits no obvious cytotoxicity toward HaCaT cells within the effective working concentration range, showing favorable cytocompatibility. In the UVA/UVB-combined induced mouse skin photoaging model, Pae-CD/gel obviously alleviates macroscopic photoaging lesions including skin dryness, erythema and wrinkles, inhibits abnormal epidermal hyperplasia, and restores dermal collagen density and ordered fiber arrangement. The in vivo restorative effect is mediated by multiple regulatory pathways: Pae-CD/gel alleviates oxidative stress damage (reducing MDA levels and recovering SOD, CAT activities and GSH contents), suppresses the secretion of pro-inflammatory cytokines (including IL-1β, IL-6 and TNF-α), and rebalances collagen metabolism, as evidenced by the upregulation of COL-I, TGF-β and hydroxyproline expression as well as the downregulation of MMP-1 and MMP-3 expression. Systematic safety evaluation verifies that Pae-CD/gel causes no significant toxic effects on major organs, hematological parameters, and liver and kidney functions at the tested administration dose. In summary, benefiting from the synergistic effect of cyclodextrin solubilization and sustained skin retention afforded by the hydrogel, Pae-CD/gel exerts potent anti-photoaging efficacy through multiple pathways involving antioxidation, anti-inflammation and collagen remodeling. This formulation provides a safe and effective transdermal delivery strategy for the treatment of skin photoaging with promising translational potential. Furthermore, the hydrogel is fabricated under mild preparation conditions, and all excipients are commercially available, which facilitates subsequent scale-up production and further clinical development.

## 4. Materials and Methods

### 4.1. Experimental Materials

Pae was purchased from Shanghai Yi’en Chemical Technology Co., Ltd. (Shanghai, China); HP-β-CD and triethanolamine (analytical grade) were purchased from Shanghai Aladdin Biochemical Technology Co., Ltd. (Shanghai, China); CMCS, Carb940, ABTS, potassium persulfate (analytical grade), and DiR fluorescent dye were purchased from Shanghai Macklin Biochemical Technology Co., Ltd. (Shanghai, China); anhydrous ethanol (analytical grade) was purchased from Sinopharm Chemical Reagent Co., Ltd. (Shanghai, China); SOD and GSH assay kits were purchased from Beijing Solarbio Science & Technology Co., Ltd. (Beijing, China); an MDA assay kit was purchased from Lianyungang 149 Biotechnology Co., Ltd. (Lianyungang, China); mouse IL-6, IL-1β, TNF-α, CAT, and TGF-β ELISA kits were purchased from Quanzhou Ruixin Biological Technology Co., Ltd. (Quanzhou, China); H_2_O_2_, PBS, and Triton X-100 were purchased from Hefei Baishark Biotechnology Co., Ltd. (Hefei, China).

### 4.2. Experimental Animals and Cells

The human immortalized keratinocyte cell line HaCaT was purchased from Shanghai Fuheng Biotechnology Co., Ltd. (Shanghai, China). DMEM high-glucose medium, penicillin/streptomycin solution, and 0.25% trypsin-EDTA digestion solution were purchased from Gibco (Waltham, MA, USA); FBS was purchased from Newzerum (Christchurch, New Zealand); the CCK-8 assay kit was purchased from Hefei Lanjieke Technology Co., Ltd. (Hefei, China); PBS buffer was purchased from Qingdao Hope Bio-Technology Co., Ltd. (Qingdao, China); and HEPES buffer was purchased from Shanghai Beyotime Biotechnology Co., Ltd. (Shanghai, China).

Male SPF-grade Kunming mice, 8 weeks old and weighing 20 ± 2 g, were purchased from the Laboratory Animal Center of Anhui University of Chinese Medicine (Hefei, China). The mice were housed in an SPF-grade barrier facility at 25 ± 2 °C and 50 ± 5% relative humidity under a 12 h/12 h light/dark cycle, with free access to food and water. All animal experiments in this study were conducted in accordance with the relevant guidelines of the Laboratory Animal Ethics Committee of Anhui University of Chinese Medicine, with the ethical approval number AHUCM-mouse-2025020.

### 4.3. Preparation and Process Optimization of Pae-CD

Pae-CD was prepared using an ultrasound-assisted saturated aqueous solution method [40]. Briefly, HP-β-CD (4.0 g) was dissolved in 20 mL of ultrapure water in a stoppered conical flask under magnetic stirring at 40 °C. Meanwhile, Pae was dissolved in ethanol with brief sonication and then added dropwise to the aqueous HP-β-CD solution under continuous stirring at 40 °C. The resulting suspension was ultrasonicated using a laboratory ultrasonic water bath (Model KH5200DE, Kunshan Hechuang Ultrasonic Instruments Co., Ltd., Kunshan, China; ultrasonic power: 200 W, frequency: 40 kHz) at designated power, temperature, and duration. After the reaction, the mixture was cooled to room temperature and stored at 4 °C overnight (12 h) to precipitate uncomplexed Pae. The precipitate was removed by centrifugation (3000× *g*, 15 min) followed by filtration through a 0.45 μm membrane filter. The filtrate was lyophilized for 48 h to obtain the solid Pae-CD complex, which was hermetically sealed and stored in the dark at 4 °C.

RSM was used to optimize the preparation process of Pae-CD [41]. Guided by preliminary single-factor evaluations, three critical processing factors—host–guest mass ratio, ultrasonic temperature, and ultrasonic time—were designated as independent variables, while encapsulation efficiency (EE, %) served as the evaluation response. Design-Expert 13.0 software was applied for polynomial regression fitting and analysis of variance (ANOVA) to assess factor interactions, model validity, and predictive capability. Confirmatory trials were subsequently conducted under the predicted optimal parameters.

### 4.4. Physicochemical Characterization of Pae-CD

The physicochemical properties of Pae-CD prepared under optimal conditions were characterized, using Pae, HP-β-CD, and their PM as comparison controls. Mean particle size, PDI, and Zeta potential were determined with a Zetasizer system (ZEN3690, Malvern Instruments, Malvern, UK). Prior to analysis, samples were appropriately diluted in ultrapure water and equilibrated at 25 °C for 2 min. Each testing procedure was conducted in triplicate.

XRD analysis was performed using an XRD (SmartLab, Rigaku, Tokyo, Japan) [42]. The testing conditions were as follows: Cu-Kα radiation source, tube voltage of 40 kV, tube current of 40 mA, scanning range of 2θ = 5–50°, scanning speed of 5°/min, and step size of 0.02°.

DSC analysis was performed using a DSC 300 instrument (NETZSCH, Selb, Germany) [43]. Briefly, 3–5 mg of each specimen was weighed into an aluminum crucible and hermetically sealed, referenced against an empty crucible. The thermal scans were run from 30 °C to 250 °C at a constant heating rate of 10 °C/min under a steady nitrogen purge (50 mL/min).

ATR-FTIR spectra were collected using an FTIR spectrometer (INVENIO S, Bruker Optics GmbH, Ettlingen, Germany) over the scanning range of 4000–500 cm^−1^ [44].

### 4.5. Preparation of Pae-CD/Gel

To prepare the matrix precursor, 0.5 g Carb940 powder was gradually sprinkled into an appropriate volume of ultrapure water and magnetically stirred until fully dispersed. Ultrapure water was supplemented to a total mass of 100 g to obtain a 0.5% (*w*/*w*) Carb940 stock solution, which was hydrated overnight at room temperature. A 2% (*w*/*w*) CMCS solution was prepared via the same procedure. CMCS and Carb940 solutions were mixed at volumetric ratios of 1:1, 1:2 and 2:1 (*v*/*v*) with continuous stirring at 300 rpm for 5 min. Afterwards, 10% (*w*/*v*) triethanolamine solution was added dropwise to adjust the pH of the mixture to 6.8 ± 0.1 for gelation initiation. Ultrapure water was supplemented to reach a total mass of 100 g, followed by another 20 min stirring to obtain blank hydrogels with different matrix ratios. Hydrogel-forming performance, 24 h swelling ratio, and rheological behaviors were taken as key indicators to screen the optimal matrix proportion.

With the optimal matrix ratio fixed, Pae-CD/gel was further fabricated. Lyophilized Pae-CD powder prepared under the optimized conditions in Section 2.3 was dissolved in ultrapure water to prepare a 10% (*w*/*v*) Pae-CD stock solution. Prior to network crosslinking, different volumes of 10% Pae-CD stock solution were blended into the uncrosslinked CMCS/Carb940 precursor mixture to achieve final Pae concentrations of 2, 4 and 6 mg/g. The liquid precursor was stirred at 300 rpm to realize homogeneous distribution of Pae-CD in the aqueous phase. Then 10% (*w*/*v*) triethanolamine solution was added dropwise to adjust pH to 6.8 ± 0.1 and trigger neutralization-mediated in situ crosslinking. Ultrapure water was supplemented to adjust the total mass to 100 g, and stirring was maintained for 20 min to acquire homogeneous Pae-CD/gel with various drug loadings. In vitro cumulative release profiles were adopted as the major criterion to determine the optimal drug loading.

### 4.6. Performance Characterization and Formulation Optimization of the Composite Gel

#### 4.6.1. Screening of the Composite Gel Matrix

To investigate how varying CMCS/Carb940 proportions influence the architecture and functional features of drug-free composite gels, their micromorphological features, water uptake capability, and viscoelastic performance were systemically assessed. Blank hydrogel formulations prepared at diverse ratios were subjected to pre-freezing at −40 °C for 12 h, followed by freeze-drying over 48 h. After gold sputter coating, the lyophilized samples were observed using field-emission scanning electron microscopy (Apreo 2S, Thermo Fisher Scientific, Waltham, MA, USA) to examine their microscopic pore structures [45].

The swelling ratio of the lyophilized gels was determined using a gravimetric method [46]. The initial weight of each freeze-dried specimen was recorded as *W*_0_, after which the sample was fully submerged in 50 mL of PBS solution (pH 7.4) and maintained at 37 °C for 24 h. Upon removal, non-absorbed surface liquid was carefully blotted away using filter paper, and the swollen mass was measured as *W_t_*. The swelling index was calculated according to the following equation:

$$\mathrm{SR}\;(\%)=\frac{w_{t}-w_{0}}{w_{0}}\times 100\%$$

The rheological properties of gels with different ratios were measured using a Discovery HR2 rotational rheometer (DHR2, TA Instruments, New Castle, DE, USA) [47]. Assays were conducted employing a 40 mm parallel-plate geometry set to a gap width of 1 mm, with experimental conditions held steady at 25 °C. Dynamic frequency sweeps were executed within the linear viscoelastic region under a fixed 1% strain across 0.1–10 Hz, tracking the storage modulus (G′) and loss modulus (G″) profiles as a function of oscillation frequency.

#### 4.6.2. Screening of Drug Loading in the Drug-Loaded Gel

After determining the optimal matrix ratio, the in vitro release behavior of Pae-CD/gel with different Pae concentrations (2, 4, and 6 mg/g) was evaluated using the dialysis bag method [48]. After determining the optimal matrix ratio, the in vitro release behavior of Pae-CD/gel with different Pae concentrations (2, 4, and 6 mg/g) was evaluated using the dialysis bag method [48]. Prior to testing, dialysis tubing with a molecular weight cutoff (MWCO) of 3500 Da was pre-conditioned by soaking in ultrapure water for 24 h. Accurately weighed samples of each drug-loaded formulation (2.0 g) were transferred into individual dialysis bags and securely tied at both ends. The assemblies were immersed into conical flasks containing 100 mL of PBS (pH 7.4) and incubated under continuous agitation (100 rpm) at 37 °C. At specified time intervals, 2 mL aliquots of the dissolution medium were sampled and immediately replaced with equal volumes of fresh PBS pre-warmed to 37 °C. Absorbance readings were recorded at 274 nm using a UV-Vis spectrophotometer, from which Pae concentrations were derived based on the calibration curve. The percentage of cumulative drug release (Rn, %) was calculated according to the following expression:

$$R_{n}\;(\%)=\frac{C_{n}\times V+\sum_{i=1}^{n-1}C_{i}\times V_{i}}{M_{0}}\times 100\%$$

where Rn (%) denotes the cumulative release percentage of Pae; Cn is the measured Pae concentration in the dissolution medium at the n-th sampling point; V corresponds to the total release medium volume (100 mL); Ci reflects the Pae concentration at the i-th sampling interval; Vi signifies the sampling volume withdrawn (2 mL); and M0 indicates the theoretical total mass of Pae initially loaded into the gel within the dialysis bag.

### 4.7. Evaluation of In Vitro Antioxidant Activity

The in vitro antioxidant activity of each formulation was evaluated using the ABTS radical scavenging assay [49]. Briefly, 7.4 mmol/L ABTS solution was reacted with an equivalent volume of 2.6 mmol/L potassium persulfate in the dark at room temperature for 12 h to generate the ABTS^+^ stock solution. Prior to experimentation, the stock solution was diluted with PBS (pH 7.4) until the absorbance at 734 nm was adjusted to 0.70 ± 0.02, obtaining the ABTS^+^ working solution.

Free Pae was initially dissolved in a minimal volume of anhydrous ethanol and further diluted with PBS (final ethanol content <1%). Pae-CD was directly solubilized in PBS. For gel samples, Pae-CD/gel was extracted in PBS at 37 °C for 24 h at a concentration of 0.1 g/mL, whereas Pae-gel was extracted under identical conditions using PBS supplemented with 1% anhydrous ethanol. Following extraction, all mixtures were centrifuged (3000× *g*, 10 min), and the resulting supernatants were passed through 0.45 μm microporous membranes and diluted to equivalent Pae concentrations using corresponding media. Subsequently, 0.1 mL of each sample solution was added to 3.9 mL of the ABTS^+^ working solution and incubated in the dark at ambient temperature for 6 min. Absorbance values were measured at 734 nm. The free radical scavenging percentage was calculated as follows:

$$Scavenging\;rate\;\left(\%\right)=\left(1-\frac{A_{S}-A_{C}}{A_{0}}\right)\times 100\%$$

where A_0_ represents the absorbance of the blank control group, As represents the absorbance of the reaction mixture containing the test sample and radicals, and Ac represents the absorbance of the background sample control.

### 4.8. Evaluation of Cell Safety Using the CCK-8 Assay

The biosafety of Pae and Pae-CD/gel in HaCaT cells was evaluated using the CCK-8 assay [50]. Pae-CD/gel was added to DMEM complete medium containing 10% FBS at a ratio of 0.1 g/mL and extracted by shaking at 37 °C and 100 rpm for 24 h. The extract was sterilized through a 0.22 μm microporous membrane and used as the stock solution. Pae was dissolved with the aid of a small amount of anhydrous ethanol and prepared into a series of concentrations using DMEM complete medium containing 10% FBS, with the final ethanol concentration not exceeding 1%. Medium containing 1% anhydrous ethanol was used as the solvent control.

HaCaT cells in the logarithmic growth phase were seeded into 96-well plates at a density of 1 × 10^4^ cells/well and cultured in a 37 °C incubator with 5% CO_2_ for 24 h. After cell attachment, the original medium was discarded, and different concentrations of blank gel extract, Pae solution, and Pae-CD/gel extract were added, respectively. Five replicate wells were set for each group, with blank control and solvent control groups included. After further incubation for 24 h, 10 μL of CCK-8 working solution was added to each well, followed by incubation in the dark for 2 h. The absorbance was measured at 450 nm, and the relative cell viability was calculated.

### 4.9. In Vitro Transdermal Permeation Study

The in vitro transdermal permeation behavior of Pae from Pae solution, Pae-CD solution, Pae-gel, and Pae-CD/gel was evaluated using the Franz diffusion cell method [51]. Fresh mouse dorsal skin was collected, and the subcutaneous adipose tissue was re-moved. The skin was rinsed three times with normal saline and examined to confirm that it was intact and undamaged. The skin was then fixed between the diffusion cells, with the stratum corneum facing the donor chamber and the dermis facing the receptor chamber. The receptor chamber was filled with PBS at pH 7.4 preheated to 32 ± 0.5 °C, and the system was equilibrated in a constant-temperature water bath shaker at 32 ± 0.5 °C and 100 rpm for 30 min.

Once equilibration finished, 2 mL of each sample formulation was loaded into the donor chamber and uniformly distributed on the skin surface. At scheduled time intervals, 2 mL receptor solution was sampled, and an identical volume of preheated fresh PBS was supplemented immediately. A UV–visible spectrophotometer was used to detect absorbance at 274 nm. The standard calibration curve was adopted to calculate Pae concentration as well as cumulative penetrated quantity. The cumulative permeation amount per unit area was calculated via the equation shown below:

$$Q_{n}=\frac{C_{n}\times V+\sum_{i=1}^{n-1}C_{i}\times V_{i}}{S}$$

where Cn represents the Pae concentration in receptor medium collected at the nth sampling time; V_i_ is the total liquid volume of the receptor chamber; Ci denotes the Pae concentration measured at the ith sampling time; V_i_ is the sampling volume taken each time; and S stands for the effective diffusion area of the Franz cell.

### 4.10. Evaluation of Skin Adhesion and Sweat-Resistance Performance

The skin retention capacity and sweat-rinse resistance of the formulations were evaluated using DiR fluorescence tracing. An appropriate amount of DiR was dissolved in anhydrous ethanol to prepare the DiR control solution. Pae-CD was labeled with DiR to obtain the Pae-CD-DiR solution. Pae-CD-DiR was then dispersed in the CMCS–Carb940 composite gel matrix according to the optimized formulation ratio to prepare Pae-CD/gel-DiR. The final DiR concentration was kept consistent among all groups.

Male SPF-grade KM mice were used. At 24 h before the experiment, dorsal hair was removed using depilatory cream, and the skin was examined to confirm that it was intact and undamaged. The mice were randomly divided into three groups. Equal amounts of DiR control solution, Pae-CD-DiR solution, and Pae-CD/gel-DiR were evenly applied to the designated dorsal areas. Initial fluorescence images were acquired using an IVIS Lumina III small-animal in vivo imaging system, and the initial fluorescence intensity was quantitatively analyzed.

At 30 min after application, artificial sweat was uniformly sprayed onto the administration area at a dose of 100 μL/cm^2^. The artificial sweat consisted of 0.5% NaCl, 0.1% urea, and 0.1% lactic acid, with the pH adjusted to 6.6 using dilute ammonia solution. After standing at room temperature for 30 min, fluorescence images were acquired again. The mean radiant efficiency within the same region of interest (ROI) was analyzed using Living Image 4.4 software, and the skin retention rate was calculated.

### 4.11. Animal Grouping and Establishment of the Photoaging Model

After 1 week of acclimatization, male SPF-grade KM mice were randomly allocated into six groups via random number table, with 10 animals per group: blank control group, UV model group, Pae group, Pae-CD group, Pae-gel group, and Pae-CD/gel group. Prior to model construction, dorsal hair of mice was depilated, and skin integrity was visually verified to exclude mechanical damage. All groups except the blank control were subjected to simultaneous UVA and UVB exposure for the construction of a chronic skin photoaging model [52]. The irradiation procedure continued for 8 weeks, and irradiation treatment was carried out once every two days. For weeks 1–2, the UVA dosage was set at 1200 mJ/cm^2^ and UVB at 1 minimal erythema dose (MED); during weeks 3–5, UVA was elevated to 2400 mJ/cm^2^ and UVB to 2 MED; from week 6 to week 8, the UVA dose was further increased to 3600 mJ/cm^2^, accompanied by UVB dose adjusted to 3 MED.

Starting from week 3, corresponding formulations were evenly spread onto the depilated dorsal region of mice in each administration group once daily at an application dose of 4 mg/cm^2^ until week 8. The Pae content in all therapeutic preparations was unified to 4 mg/g to guarantee consistent drug dosage. Equal volumes of normal saline were applied to mice of blank control and UV model groups. Throughout the experiment, macroscopic alterations of dorsal skin such as erythema, desquamation and wrinkles were observed and documented at fixed weekly time points.

All animals received isoflurane anesthesia 24 h following the final topical administration at week 8, and 0.8–1.0 mL whole blood was harvested via retro-orbital venous plexus bleeding [53]. Blood samples were placed at room temperature for 30 min, followed by centrifugation at 3000 rpm for 15 min under 4 °C to isolate serum for subsequent biochemical detection. After blood sampling, mice were sacrificed via cervical dislocation. Full-thickness dorsal skin within the depilated region was rapidly dissected. Partial skin tissues were fixed in 10% neutral formalin over 24 h, then subjected to gradient dehydration, transparency, paraffin embedding, and cut into 4 μm slices for histopathological staining and immunohistochemical testing. The leftover skin specimens were homogenized with pre-cooled normal saline at a proportion of 1:9 (*w*/*v*) in ice bath, and centrifuged at 12,000 rpm for 15 min at 4 °C. The collected supernatants were used to determine oxidative stress markers, inflammatory cytokines and collagen-associated indexes.

### 4.12. Histopathological Staining of Skin Tissue

Paraffin sections were subjected to H&E staining, Masson’s trichrome staining, and Picrosirius Red staining, respectively [54]. Skin tissue morphology, collagen fiber distribution, and collagen content were observed under a light microscope. ImageJ software (Version 1.54r) was used to quantitatively analyze parameters such as epidermal thickness and collagen volume fraction.

### 4.13. Immunohistochemical Analysis

Immunohistochemistry (IHC) was carried out to detect the expression levels of MMP-3 and COL-1 proteins in skin samples. Paraffin slices were baked at 60 °C for 60 min, deparaffinized with xylene, and rehydrated via gradient ethanol solutions. Antigen retrieval was performed using pH 9.0 EDTA buffer and pH 6.0 sodium citrate buffer separately. Subsequently, sections were incubated with 3% H_2_O_2_ at room temperature away from light for 15 min to eliminate endogenous peroxidase activity. After rinsing with PBS-T, 5% goat serum was used to block non-specific binding at room temperature for 20 min. Diluted primary antibodies (1:200) were added, followed by incubation at 37 °C for 60 min. After washing steps, HRP-conjugated secondary antibodies were supplemented and incubated at 37 °C for 30 min. The slices were visualized with DAB chromogen, counterstained with hematoxylin, dehydrated, cleared, and mounted with neutral balsam. Microscopic images were captured under a light microscope. ImageJ software (Version 1.54r) was utilized to measure the average optical density (AOD) of positive staining for semi-quantitative evaluation of target protein expression.

### 4.14. Determination of Biochemical Indicators in Skin Tissue

#### 4.14.1. Detection of Oxidative Stress Indicators

The supernatant of skin tissue homogenates was collected, and SOD activity, as well as the levels of MDA, GSH, and CAT, were measured according to the instructions of the corresponding assay kits.

#### 4.14.2. Determination of HYP Content

The supernatant of skin tissue homogenates was collected, and HYP content was determined according to the instructions of the corresponding assay kit to reflect the total collagen level in the skin.

#### 4.14.3. Determination of Inflammatory Factors and MMP-1 Content

The supernatant of skin tissue homogenates was collected, and the levels of IL-1β, IL-6, TNF-α, and MMP-1 were determined using ELISA in strict accordance with the instructions of the corresponding kits. The absorbance of each well was measured at 450 nm, and the contents of the target indicators were calculated according to the standard curves.

### 4.15. Routine Blood Test and Liver and Kidney Function Assessment

Routine blood parameters, including WBC, RBC, HGB, and PLT, were measured in EDTA-anticoagulated whole blood using an automated hematology analyzer. Serum levels of ALT, AST, BUN, and Cr were measured using an automated biochemical analyzer to comprehensively evaluate the systemic safety of the topical formulations.

### 4.16. Statistical Analysis

All data analyses were performed using GraphPad Prism software (version 10.1.2, GraphPad Software, LLC, San Diego, CA, USA). Experimental data are uniformly expressed as the mean ± standard deviation (SD). Differences between two independent groups were assessed via a two-tailed unpaired Student’s *t*-test. For multiple-group comparisons, one-way or two-way analysis of variance (ANOVA) followed by Sidak’s post hoc multiple comparisons test was applied according to the experimental design. Statistical significance was defined as *p* < 0.05 (* *p* < 0.05, ** *p* < 0.01, *** *p* < 0.001).

## Data Availability

The data presented in this study are openly available in the article.
